# Game based assessments of cognitive ability in recruitment: Validity, fairness and test-taking experience

**DOI:** 10.3389/fpsyg.2022.942662

**Published:** 2023-01-18

**Authors:** Franziska Leutner, Sonia-Cristina Codreanu, Suzanne Brink, Theodoros Bitsakis

**Affiliations:** ^1^Institute of Management Studies, Goldsmiths, University of London, London, United Kingdom; ^2^HireVue, Inc, Salt Lake City, UT, United States; ^3^King’s College London, London, United Kingdom

**Keywords:** game based assessment, cognitive ability, adverse impact, user experience, outcome parity, fairness, psychometric tests, recruitment

## Abstract

Gamification and machine learning are emergent technologies in recruitment, promising to improve the user experience and fairness of assessments. We test this by validating a game based assessment of cognitive ability with a machine learning based scoring algorithm optimised for validity and fairness. We use applied data from 11,574 assessment completions. The assessment has convergent validity (*r* = 0.5) and test–retest reliability (*r* = 0.68). It maintains fairness in a separate sample of 3,107 job applicants, showing that fairness-optimised machine learning can improve outcome parity issues with cognitive ability tests in recruitment settings. We show that there are no significant gender differences in test taking anxiety resulting from the games, and that anxiety does not directly predict game performance, supporting the notion that game based assessments help with test taking anxiety. Interactions between anxiety, gender and performance are explored. Feedback from 4,778 job applicants reveals a Net Promoter score of 58, indicating more applicants support than dislike the assessment, and that games deliver a positive applicant experience in practise. Satisfaction with the format is high, but applicants raise face validity concerns over the abstract games. We encourage the use of gamification and machine learning to improve the fairness and user experience of psychometric tests.

## 1. Introduction

Cognitive ability tests have been used as popular selection and recruitment tools for decades, with good reason: The predictive validity of cognitive ability for job performance is well documented. A meta analytic review of over 20,000 studies combining 5 million participants shows an average correlation of *r* = 0.5 between cognitive validity and job performance (Kuncel et al., 2010). Cognitive ability is related to a variety of performance outcomes, including supervisor rated performance (*r* = 0.42–0.57, *p* < 0.001; Judge et al., 1999) and extrinsic career success (*r* = 0.53, *p* < 0.001; Higgins et al., 2007). In addition, cognitive ability tests are amongst the most effective selection methods available: Schmidt and Hunter’s review of meta-analytic studies on 19 selection methods reports a predictive validity of intelligence of *r* = 0.51 for job performance, rivalled only by work sample tests (*r* = 0.54) and structured interviews (*r* = 0.51) (Schmidt and Hunter, 1998). In addition, cognitive ability predicts a wide range of real world outcomes such as educational attainment, socioeconomic status, and career success (Schmidt and Hunter, 2004).

In recent years, gamification of cognitive tests has become a popular method for improving the test taker experience of psychometric tests (Raghavan et al., 2020). Gamification entails the introduction of game mechanisms as motivational affordances designed to result in psychological outcomes such as motivation, attitude and enjoyment (Hamari et al., 2014). In the context of recruitment, gamification should also include a level of real world connection and job relatedness (Korn et al., 2017). Game mechanisms include adaptive levels, progression, immediate feedback, and intermittent goals (Tremblay et al., 2010; Landers and Callan, 2011; Palmer et al., 2012). In the psychometric assessment for recruitment context, gamification also implies optimisation for mobile and online use (Landers and Callan, 2011). Gamification is studied in vastly different areas from entertainment and video games to gamified work tasks, but the most common context is serious games for learning (Hamari et al., 2014) or, within organisational psychology, gamification for employee training or customer engagement (Tippins, 2015).

Descriptions of gamified cognitive ability tests for use as psychometric assessments in recruitment are rare in the literature. However, several studies describe gamified measures of cognitive abilities that approximate performance on established, non-gamified tasks. For example, a gamified four-dimensional spatial task predicts performance on several cognitive ability tasks including a working memory span task (*r* = 0.62, *p* < 0.05), a quantitative reasoning task (*r* = 0.30, *p* < 0.05), and the Raven’s progressive matrices, a popular abstract reasoning test (*r* = 0.37, *p* < 0.05) (Atkins et al., 2014). In a similar vein, gamified versions of working memory and processing speed tasks correlate with the Raven’s Progressive Matrices short form between *r* = 0.29 (*p* < 0.01) and *r* = 0.45 (*p* < 0.05) (McPherson and Burns, 2008). Cognitive tasks implemented in the computer game Minecraft have high convergent validity with both Raven’s Standard Progressive Matrices and the Vandenberg & Kuse Mental Rotations Test (*r* = 0.72, *p* < 0.002 in Peters et al., 1995, 2021; Raven, 2000). Gameplay on 12 short video games selected specifically to test components of intelligence correlated strongly with cognitive ability assessed across 11 established cognitive ability tests (*r* = 0.93, *p* < 0.01; Quiroga et al., 2015).

Additional studies describe game based cognitive ability tests developed for specific populations such as cognitive testing and training of children (Verhaegh et al., 2013), detection of cognitive decline in elders (Jimison et al., 2004, 2007), entertainment games (Gamberini et al., 2010), or edutainment games to train cognitive ability (Dandashi et al., 2015). Some evidence for the predictive validity of gamified tasks is provided by two studies on school children. Performance on gamified versions of working memory and processing speed tasks correlates similarly with school performance and performance on the Raven’s matrices (McPherson and Burns, 2008). A game-like cognitive task differentiates between students with typical and low mathematics achievement (AUC = 0.897; Luft et al., 2013).

### 1.1. The gamification advantage

Entertainment games deliver a range of behavioural and affective outcomes (Connolly et al., 2012). These include immersion (Jennett et al., 2008) and experiences of flow (Chen, 2007; Nacke et al., 2009), which in turn enhance enjoyment (Weibel et al., 2008). The goal of gamification is to produce the same subjective experiences as games do (Huotari and Hamari, 2012), and to use game mechanisms to create enjoyment and playability (Landers and Callan, 2011). Indeed, the majority of studies that describe gamification observe positive effects on players’ subjective experiences, including on motivation, attitude and enjoyment, indicating that gamification is successful at producing some of the same affective outcomes as entertainment games (Hamari et al., 2014). For example, gamified tasks increase attention (Klein et al., 2017) and cognitive test performance (Lumsden et al., 2016). Market research questionnaires that are gamified increase enjoyment compared to those that are not (Guin et al., 2012). In addition to game mechanics, framing a task as a game alone increases interest and enjoyment (Lieberoth, 2015). Introducing gamification elements into HR tasks such as onboarding increases enjoyment relative to non-gamified tasks (Heimburger et al., 2020).

In the context of assessment, gamification might also help reduce test taking anxiety, which could in turn improve performance. Game based assessments might help reduce the effect of moderators and mediators of differences in test performance, namely test anxiety and stereotype threat. Test anxiety is an unpleasant emotional state experienced in testing situations (Dusek, 1980). Gamification provides users with a less anxiety-inducing environment by unobtrusively logging behaviour in a “fun” way, allowing the participant to fully immerse in the task and making it feel less threatening (McPherson and Burns, 2008; Kato and de Klerk, 2017). This decrease in anxiety can lead to increased performance compared to traditional pen-and-paper tests. For example, participants completing a gamified version of a multimedia systems knowledge test have decreased anxiety and increased performance compared to those completing a traditional paper test (Mavridis and Tsiatsos, 2017). Furthermore, gamification reduces performance differences caused by stereotype threat, with no gender performance differences observed in a high threat gamified logic test (Albuquerque et al., 2017).

### 1.2. Fairness and diversity problems in cognitive ability tests

In application for recruitment and selection, cognitive ability tests are prone to generating adverse impact for protected applicant groups (Hunter and Hunter, 1984). Compared to 16 of the most common selection methods, general mental ability tests generate the highest group differences between ethnicities (Ployhart and Holtz, 2008). These group differences disadvantage minority applicants in particular, thereby limiting the benefits of using cognitive ability tests as a pre-selection tool for several reasons: First, the use of potentially biassed selection methods raises ethical and human rights concerns (Yam and Skorburg, 2021). Second, although cognitive ability is related to job performance, diversity is an important hiring outcome associated with firm financial performance (Erhardt et al., 2003; Hunt et al., 2018). Any benefits of hiring for job performance might be reduced if they in turn reduce the diversity of an organisation. Third, violating adverse impact regulations that mandate thresholds for acceptable group differences in selection processes raises legal concerns for employers (U.S. Equal Employment Opportunity Commission, 2021).

One method used by employers and test providers to mitigate adverse impact of cognitive ability tests is to combine them with personality tests. This method was first proposed by Hunter and Hunter (1984). However, the method is not always effective in reducing adverse impact (Ryan et al., 1998; Avis et al., 2002). “Culture-fair” cognitive ability tests attempt to remove the impact of language, literacy and cultural values around rapid performance by removing any items that require explicit use of language, reading, or time constraints and focusing on visuospatial or abstract reasoning instead (Jensen, 1980; Arvey and Faley, 1988; Anastasi and Urbina, 1997). This, again, is not always effective, with culture-fair tests like the Raven’s Progressive Matrices producing group differences at the threshold of adverse impact in a real world selection process (LeBlanc and Chawla, 2003).

### 1.3. Machine learning based psychometric assessments

In traditional cognitive ability tests, scores are based on counting correct and incorrect answers to a number of items, typically answered within a specified time. Machine learning based scoring instead employs statistical models like regression as the scoring algorithm: Gameplay data predicts scores on an established cognitive ability test. The resulting prediction model is the scoring algorithm (e.g., see Atkins et al., 2014; Quiroga et al., 2015). This type of scoring is commonly used in the emerging field of computational psychometrics, for example to translate a person’s digital footprint into a psychometric profile (Roth et al., 2013; Kosinski et al., 2016), or to measure personality based on language use (Schwartz et al., 2013; Lambiotte and Kosinski, 2014; Park et al., 2015), video interviews (Hickman et al., 2021), or gameplay (Leutner et al., 2020). The digitalised format of game based assessments lends itself to machine learning based scoring because it allows for the collection of several gameplay behaviours such as levels completed or times spent on a single task, rather than simply recording correct or incorrect answers.

Machine learning based scoring algorithms for cognitive ability games have a distinct advantage in the applied selection context: They can be optimised for concurrent validity and, at the same time, for minimising adverse impact, a process described by several machine learning based assessment vendors (Raghavan et al., 2020). This is typically achieved by selecting features that predict the target outcome and deleting or down-weighting features that are also associated with protected group membership like age, gender, or ethnicity (Bogen and Rieke, 2018; Diamond et al., 2020; Leutner et al., 2021; Team PredictiveHire, 2021). The ability to optimise scoring models for fairness gives providers more flexibility in reducing the adverse impact of their assessment procuts, a process that in the past involved costly and resource intensive redesigning of assessments (Raghavan et al., 2020).

A common critique of machine learning models is that they are “black-boxes.” Opaque algorithms obscure what is being assessed and how it might discriminate against different applicants, impacting their human right to work (Yam and Skorburg, 2021). Yam and Skorburg (2021) describe three areas of algorithm opacity: First, the technical literacy of hiring managers who may wrongly interpret scores. This risk is minimised when machine learning based scoring is used to assess existing psychometric constructs like cognitive ability, and when assessments are compared to established measures. Second, a lack of transparency from test publishers that allows for biassed practises. This risk is minimised by peer review that allows for discussion and critique within the scientific community. Finally, representational characteristics of the machine learning models used to score assessments. This risk is reduced by using interpretable models such as regression or certain decision trees, and interpretable features, such as gameplay performance on face and content valid tasks.

### 1.4. Current limitations

There is a lack of academic studies describing game based assessments of cognitive ability as well as machine learning based assessments for the selection and recruitment context. Although these assessments are described in the literature for learning, development, education, or research contexts, the applied challenges in recruitment and selection are unique in terms of their requirements for transparency, accuracy, and fairness. Recruitment decisions impact the lives of individuals, and recruitment algorithms, or machine learning based assessments, affect applicants’ human rights (Yam and Skorburg, 2021). Crucially, the European Union’s proposed artificial intelligence regulation classes recruitment and selection algorithms as high risk systems, meaning they require regular conformity assessments and must comply with standards of transparency, validity, data management and more (European Commission, 2021).

There is a lack of data and studies on the diversity benefits of game based assessments and machine learning based scoring. Studies are needed to demonstrate whether scoring algorithms optimised for fairness are robust when adapted to different samples (Raghavan et al., 2020). Similarly, game based formats could help reduce the adverse impact of cognitive ability tests. Given studies showing that anxiety negatively impacts test performance, and that gamification reduces anxiety in the serious games and education contexts, studies are needed to explore the link between game based formats, anxiety and assessment scores in recruitment contexts (Richards et al., 2000; Thames et al., 2015; Kiili and Ketamo, 2017). Finally, user experience benefits are a main driver for the adoption of game based assessments (Tippins, 2015). Subjective user experience improvements, however, are largely context dependent (Hamari et al., 2014) and there is a lack of studies on applicant reactions to game based assessments in a job selection context to validate that gamification benefits translate to job applicants.

## 2. Materials and methods

We use data from 11,574 completed game based cognitive ability assessments, of which 10,924 come from job applicants and 650 from panellists (HireVue, 2021). Across four studies we address limitations in the current literature by describing the development, validity, fairness, and user experience of a machine learning scored, game based assessment of cognitive ability in the recruitment and selection context. In validating the game based assessment, we evaluate the potential of both gamification and machine learning based scoring to alleviate long standing problems with group differences in cognitive ability tests. We evaluate the applicant user experience with game based cognitive ability tests in a real world job application context. We are not aware of existing studies providing a comprehensive overview of a game based assessment used in the applied selection context.

### 2.1. Study 1: Developing and validating a machine learning based scoring algorithm for a game based assessment: Convergent validity, test–retest reliability

Using a sample of 3,609 panellists and real world job applicants, we develop a machine learning based scoring algorithm for recruitment and selection, based on two short games designed to measure cognitive ability. The algorithm is optimised for convergent validity with an established cognitive ability test as well as minimising group differences on age, gender, and ethnicity (ICAR; The International Cognitive Ability Resource Team, 2014). Gameplay features and scoring model weights are described to provide transparency, and we evaluate test–retest reliability.

### 2.2. Study 2: Testing adverse impact and fairness in an independent sample of 3,107 job applicants

In order to address a lack of literature on the effectiveness of machine learning based models optimised for fairness, we analyse the performance of a further 3,107 real life job applicants on the game based assessment to determine whether it leads to adverse impact on age, gender and ethnicity. We include adverse impact metrics that address outcome parity as they are typically used in psychometric test evaluation: Adverse Impact Ratio, Cohen’s *D*, and two Standard Deviations.

### 2.3. Study 3: The user experience: Test taking anxiety in game based assessments

We explore potential benefits of the game based assessment format on the user experience, and in turn assessment performance and fairness. Given that test taking anxiety can impact gender differences on cognitive ability tests, and given that gamification promises to reduce anxiety by increasing engagement (Osborne, 2007; Kato and de Klerk, 2017), we test the effect of anxiety and gender on game based assessment performance. The experimental study includes data from 85 panellists.

### 2.4. Study 4: In application: The user experience of job applicants

Given the lack of research on the user experience of gamification in the context of job applications, we review feedback from 4,778 real world job applicants who completed game based cognitive ability assessments. We discuss the implications for the claim that game based assessments deliver a user friendly experience in the job application context.

## 3. Studies

### 3.1. Study 1: Developing and validating a machine learning based scoring algorithm for a game based assessment

#### 3.1.1. Data

Two samples are used to train the scoring algorithm: The first is a scoring sample consisting of 565 panellists recruited on Prolific Academic and compensated for their participation. Participants completed the cognitive ability games as well as traditional cognitive ability tests. The second is a mitigation sample consisting of 3,044 job applicants to entry and graduate level positions advertised in the EMEA region, who were part of a group of 6,151 applications. Participants did not need to be native English speakers. Job applicants who asked for accommodations or alternative assessments are not included in the sample. The remaining 3,107 applications are retained for use in Study 2. The two samples were split approximately equally along gender, age and ethnicity to ensure a good representation of each group in each sample. The majority of applicants are leaving university to join the job market at the time of application. They complete the game based assessment as part of their application process, and also provide demographic information. The game based assessment is the first assessment step in the recruitment process, followed by further assessments and interviewing and is completed at a convenient time online. See Table 1 for descriptives.

**Table 1:** Demographics for samples used in studies one and two.

		Study 1		Study 2
		Scoring sample (*N* = 565)	Mitigation sample (*N* = 3,044)	Study two sample (*N* = 3,107)
Age	Under 40 years old	456	2,994	3,057
	Age 40 or older	109	48	49
Gender	Female	298	1,287	1,334
	Male	265	1,749	1,757
	Other	2	8	16
Ethnicity	Asian	107	816	815
	Black	91	310	309
	Hispanic	70	–	–
	Middle/Near Eastern	–	–	66
	White	297	1,918	1,917

#### 3.1.2. Measures

##### 3.1.2.1. Cognitive ability games

For the machine learning based scoring model we use gameplay data from two games developed to assess cognitive ability: Shapedance and Numerosity (described in Table 2). Gameplay tasks are modelled after traditional cognitive ability tests, and to assess aspects of cognitive ability as specified by the Cattell–Horn–Carroll theory of intelligence (McGrew, 2009). Similar to other cognitive ability tests, the playtime of each game is fixed. Several gameplay behaviour features are tracked for each game.


$$\begin{array}{l} maxLevel=\max\;levelreached\;by\;thetest-\\ \;\;\;\;\;\;\;\;\;\;\;\;\;\;\;\;\;\;\;\;\;\;\;\;\;\;\;\;takerin\;a\;game\in \left[0,100\right] \end{array}$$

$$\begin{array}{l} finalLevel=finallevelreached\;by\;thetest-\\ \;\;\;\;\;\;\;\;\;\;\;\;\;\;\;\;\;\;\;\;\;\;\;\;\;\;\;\;\;\;takerin\;a\;game\in \left[0,100\right] \end{array}$$

$$\begin{array}{l} winRatio=numberofsuccessfullevels÷\\ \;\;\;\;\;\;\;\;\;\;\;\;\;\;\;\;\;\;\;\;\;\;\;\;\;numberofmatches\in \left[0,1\right] \end{array}$$

levelsWon=numberoflevelswon

levelsLost=numberoflevelslost

$$\begin{array}{l} totalLevels=totalnumberoflevelsplayedthroughout\\ \;\;\;\;\;\;\;\;\;\;\;\;\;\;\;\;\;\;\;\;\;\;\;\;\;\;\;\;\;\;thegame\;\;\left(levelsWon+levelsLost\right) \end{array}$$

**Table 2:** Game descriptions, duration, and example screens.

Game	Duration	Description	Example
Numerosity	3 min	Mental arithmetic task where players are presented with a set of numbers and are asked to identify a combination that when summed, subtracted, multiplied or divided, yields a target result.	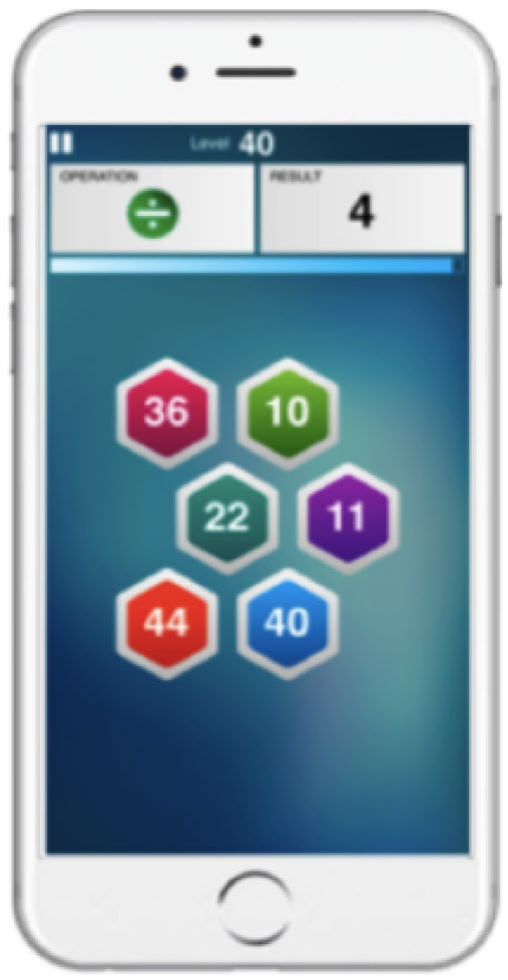
Shapedance	3.3 min	Mental rotation task that requires players to identify matching patterns of increasing complexity in rotated, and rotating, stimuli.	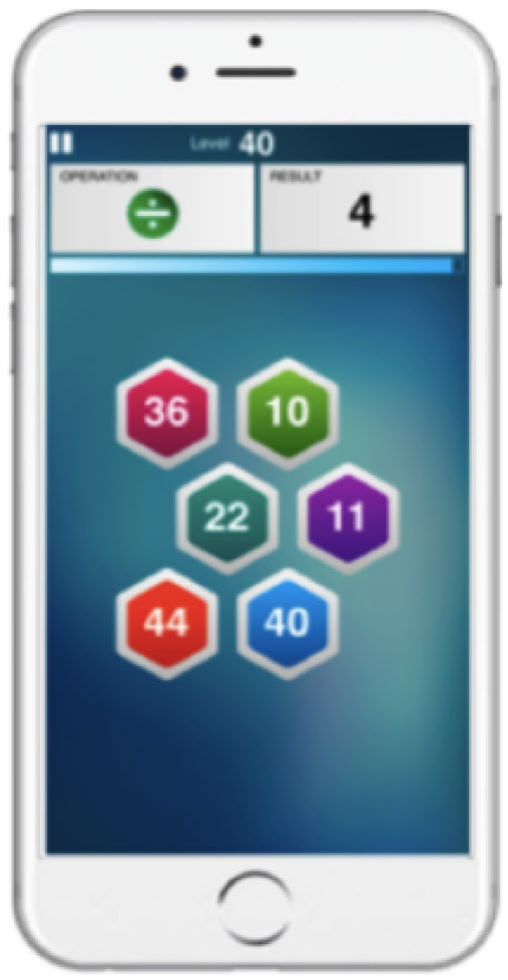
Pathfinder	5 min	Puzzle task where players move pieces in order to create a path between two endpoints.	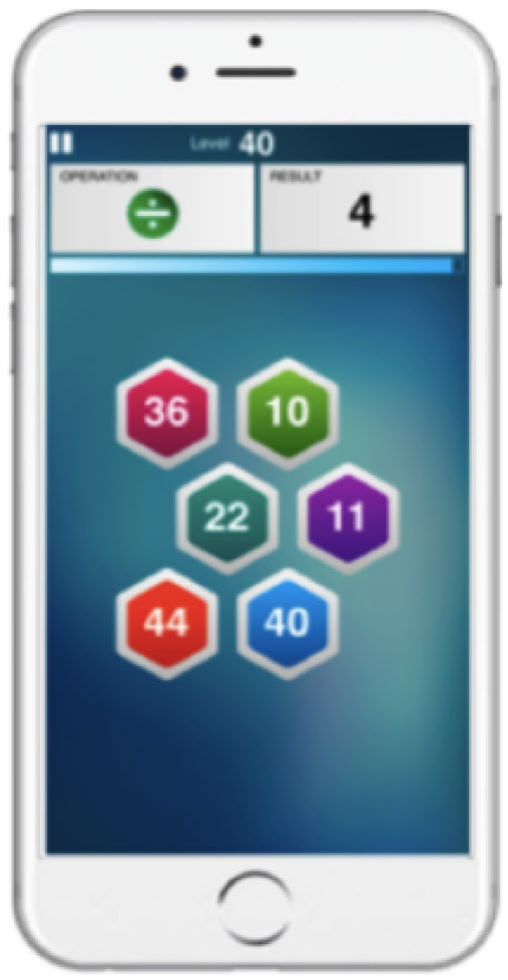
Singularity	3 min	Attention task that requires players to identify a unique shape amongst increasing numbers of distractor shapes, and with a decreasing number of unique features of the target shape.	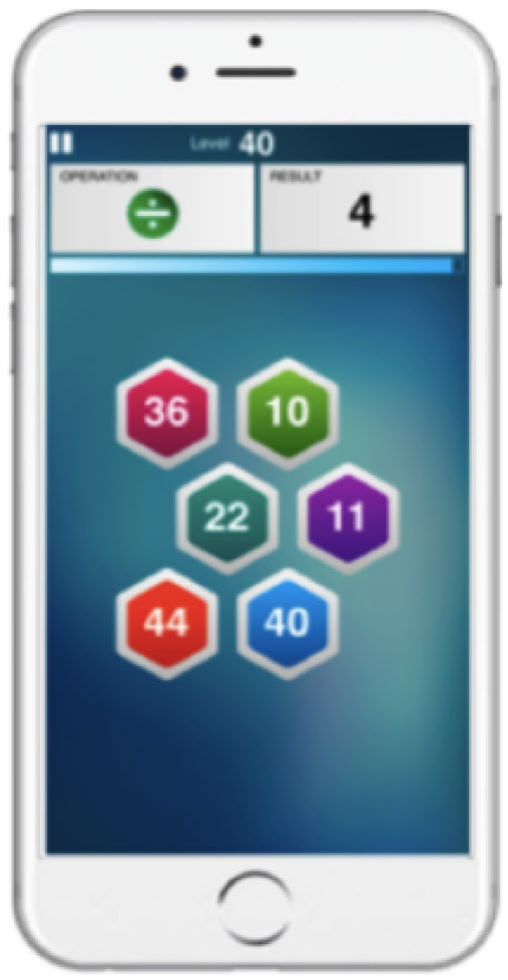

Game colours are chosen from colour blind friendly palettes. Games can be played on mobile or desktop devices. Prior to gameplay, detailed instructions are provided. Games can be accessed and played on a computer or mobile using the HireVue app (HireVue, 2021). Reproduced with permission from HireVue Inc. (www.hirevue.com).

maxLevel and finalLevel measure how far a player advances in relation to the maximum level reachable in the game (level 100 for all games). WinRatio measures how many levels are completed correctly: if the player makes no mistakes at any level, winRatio=1 . However, a player can have winRatio=1, but may not perform the task quickly enough to play many levels in the allocated time.

##### 3.1.2.2. Cognitive ability questionnaire

Cognitive ability is assessed by combining the 16-item International Cognitive Ability Resource Sample Test (ICAR; The International Cognitive Ability Resource Team, 2014) and four items from the Cognitive Reflection Test (CRT; Frederick, 2005; Toplak et al., 2014). ICAR is a public-domain assessment tool of cognitive ability, widely used in psychological and social scientific research and recommended for use in high-stakes testing, including job selection and clinical purposes. ICAR has four item types: Three-Dimensional Rotation items (four items); Letter and Number Series (four items); Matrix Reasoning (four items); and Verbal Reasoning logic questions (four items). The CRT measures deliberative/rational thinking, the tendency to override a response alternative that is incorrect, and to engage in further reflection that leads to the correct response. The CRT predicts performance on rational thinking tasks even when cognitive ability and thinking dispositions are taken into account (Frederick, 2005; Toplak et al., 2014). CRT items were included to provide a broader assessment of cognitive ability.

#### 3.1.3. Results

##### 3.1.3.1. Game based assessment scoring algorithm

To generate the scoring algorithm, gameplay behaviour features from cognitive ability games are entered into a prediction model as predictors of scores on the cognitive ability questionnaire. A variety of machine learning models with 10-fold cross validation are tested as scoring methods including Classical Regression with regularisation, Random Forests, Naive Bayes, and Support Vector Machines (Bergstra and Bengio, 2012). Ridge Regression performs best and is chosen as the scoring model. Additionally, ridge regression models have high explainability and are easily interpretable.

To minimise subgroup differences, a novel Bias Penalisation method is used to further optimise the Ridge Regression model (see Rottman et al., 2021, manuscript submitted for publication^1^). This method models the diversity-validity trade-off by adding a bias penalty term to the model optimization during the fitting process. The result is a model that minimises subgroup differences whilst maintaining high predictive accuracy. Data from both scoring and mitigation samples is used to determine penalisation within the models. The algorithm thus uses data from both samples to generate a scoring algorithm that is optimised for predicting cognitive ability as well as minimising bias.

Overall model performance is estimated by Pearson correlation between observed and predicted cognitive ability scores to allow for easy interpretability. Correlations are reported based on the aggregate of out-of-sample cross validation prediction across all folds.

##### 3.1.3.2. Descriptive statistics

Descriptive statistics for the questionnaire and game based cognitive ability assessments are presented in Table 3.

**Table 3:** Descriptive statistics for the cognitive ability questionnaire, cognitive game based assessment, and cognitive ability games features in the scoring and validation samples as well as correlations between game behaviour and cognitive ability scores.

	Scoring sample (*N* = 565)					Mitigation sample (*N* = 3,044)		
	Mean	SD	Range	Pearson’s *r*		Mean	SD	Range
				Cognitive ability questionnaire	Game based assessment			
Cognitive ability questionnaire	9.2	4.5	[0–20]			–	–	–
Game based assessment	9.2	1	[6.8–11.7]			9.6	1	[6.6–12.2]
Game features								
numerosity_finalLevel	39.4	12.4	[4–91]	0.44**	0.58**	38.7	13	[0–67]
numerosity_levelsLost	5.8	3.7	[0–25]	−0.40**	−0.56**	3.5	2.5	[0–17]
numerosity_levelsWon	22.4	5.4	[7–46]	0.36**	0.37**	21.8	5.2	[1–34]
numerosity_maxLevel	40.2	11.9	[9–91]	0.43**	0.46**	40.7	11.2	[3–69]
numerosity_totalLevels	28.2	5.9	[12–51]	0.08	0.06**	25.4	4.9	[8–35]
numerosity_winRatio	0.79	0.12	[0.38–1]	0.48**	0.59**	0.86	0.10	[0.12–1]
shapedance_finalLevel	27.1	8.9	[2–51]	0.44**	0.45**	27.6	8.5	[0–60]
shapedance_levelsLost	7.6	5	[0–30]	−0.40**	−0.81**	5.6	3.5	[0–23]
shapedance_levelsWon	16.8	3.3	[6–26]	0.28**	0.10**	16.0	3.1	[0–32]
shapedance_maxLevel	28.0	8.4	[7–51]	0.43**	0.42**	28.3	8.1	[1–60]
shapedance_totalLevels	24.5	5.6	[10–52]	−0.19**	−0.63**	21.6	4	[6–36]
shapedance_winRatio	0.70	0.14	[0.33–1]	0.45**	0.75**	0.75	0.14	[0–1]

*p  <  0.01.

##### 3.1.3.3. Model performance and concurrent validity

**Figure 1:**
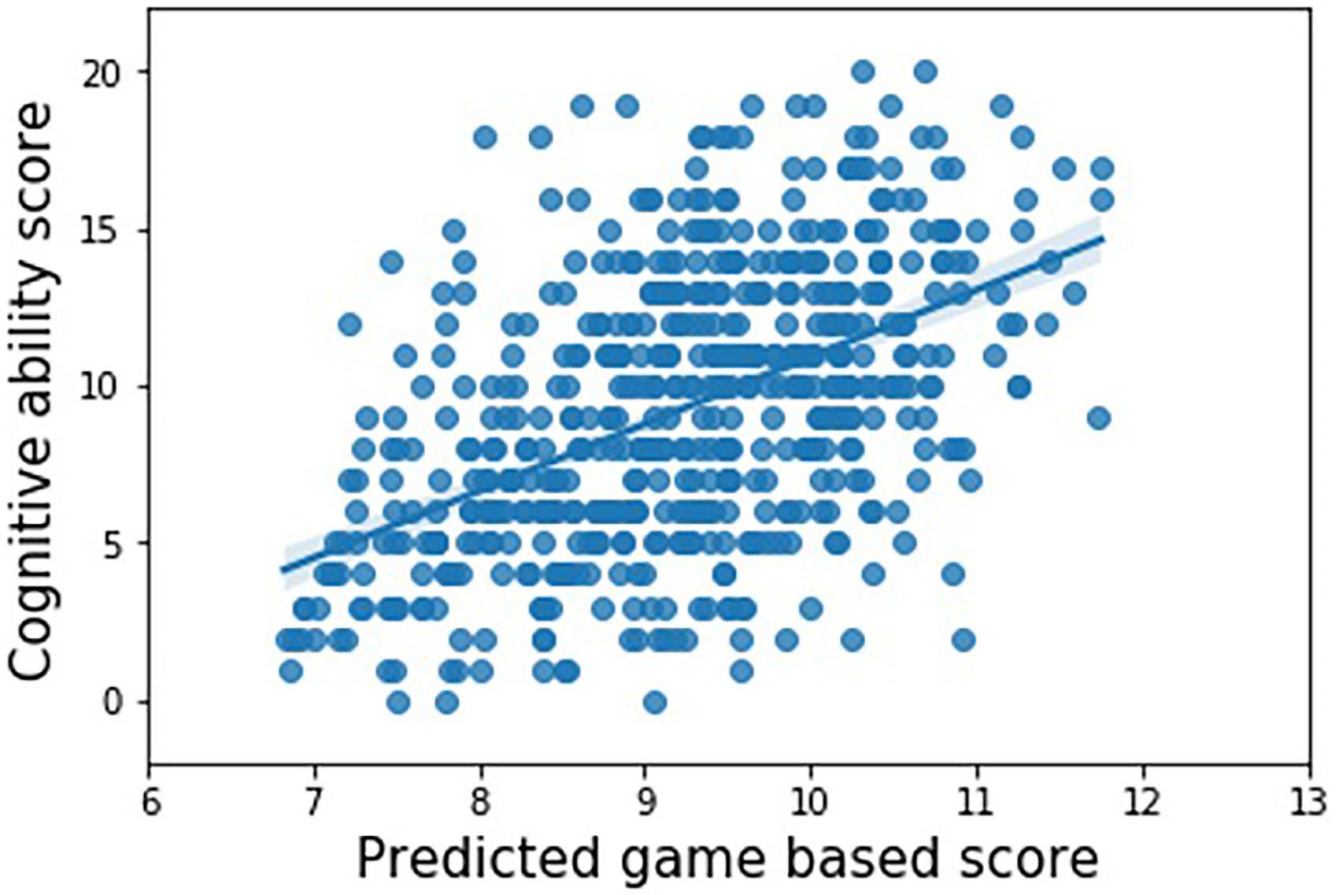
Observed questionnaire based and predicted game based cognitive ability scores using the ridge regression model with bias penalisation (Pearson’s *r* = 0.50).

The machine learning based scoring algorithm achieves a concurrent validity of *r* = 0.5 (95% CI from Fisher transformation = 0.43–0.56) with cognitive ability as measured by the cognitive ability questionnaire. See Figure 1 for an illustration of the relationship between the Game Based Assessment scores and cognitive ability scores.

##### 3.1.3.4. Explainability and feature importance

**Figure 2:**
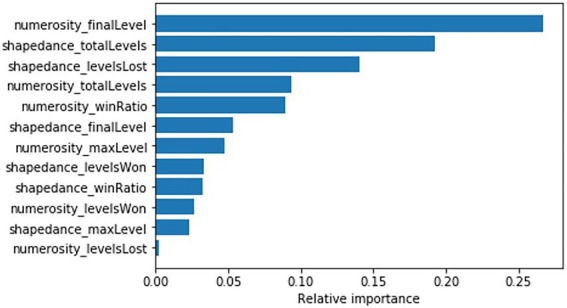
Feature relative importance for the machine learning scoring model.

Feature relative importance is computed for the scoring algorithm to determine which gameplay behaviours contribute more or less to the Game Based Assessment score (see Figure 2). Features from both cognitive ability games are represented in the scoring algorithm, with the final level reached on the Numerosity game and the total levels played on the Shapedance game being the two most important features in the scoring algorithm. Additionally, Table 3 shows the correlation between each game behaviour and cognitive ability scores on the questionnaire as well as the game based measures. Numerosity_finalLevel correlates strongly with both questionnaire and game based cognitive ability scores (*r* = 0.44 and *r* = 0.58, respectively). Shapedance_totalLevels correlates strongly with game based and moderately with questionnaire based cognitive ability (*r* = −0.19 and *r* = −0.63, respectively).

##### 3.1.3.5. Test–retest reliability

**Figure 3:**
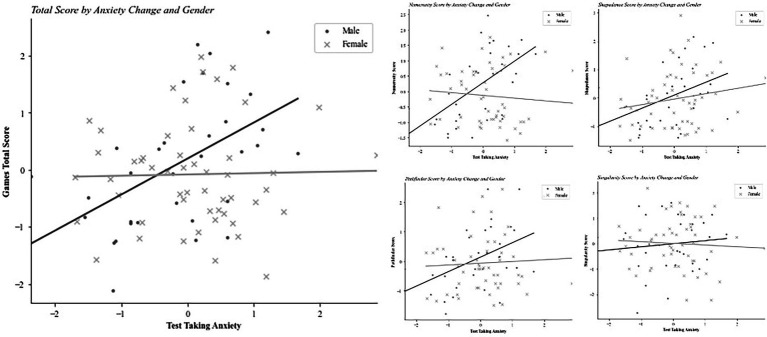
Test–retest reliability and convergent validity for the game based cognitive ability assessment.

A subset of 102 participants from the scoring sample completed the game based assessment again within 4 months of their first completion (mean age 34 with *SD* = 10.20, 53% male, 27% Asian, 20% Black, 11% Hispanic, 42% White). Test–retest reliability is computed as the correlation between assessment scores at first and second completion and is moderate to high with Pearson’s *r* = 0.68 (*p* < 0.001) (see Figure 3).

#### 3.1.4. Discussion

The convergent validity of *r* = 0.50 achieved for the game based cognitive ability test with using Bias Penalisation compares to validities described in the literature: When correlating game based with single or separate established cognitive ability measures obtain r values of around *r* = 0.30 to 0.45 (e.g., McPherson and Burns, 2008; Atkins et al., 2014). Though studies using structural equation modelling to correlate latent factors derived from several traditional tests with those derived from a range of games report higher correlations ranging from *r* = 0.72 to 0.93 (Quiroga et al., 2015; Peters et al., 2021).

Study two tests whether the scoring algorithm with Bias Penalisation performs well in practise when used on a dataset of job applicants that were not known during model training. This is important given a lack in the literature of studies describing how bias mitigation methods might behave vis a vis previously unseen samples (Raghavan et al., 2020).

### 3.2. Study 2: Testing adverse impact and fairness in an independent sample of 3,107 job applicants

We test the generalisability of the adverse impact performance of our scoring algorithm generated in Study 1. This is done by evaluating the adverse impact for a set of participants not contained in the scoring algorithm dataset. Participants are scored using the scoring algorithm generated in Study one. Adverse Impact metrics are then computed for the participants and evaluated.

#### 3.2.1. Data

We use data from 3,107 further job applicants to graduate and entry level roles, as described in Study one. All applicants complete the game based assessment with Shapedance and Numerosity that we describe in Study one.

#### 3.2.2. Method

Adverse impact and group differences are tested using several metrics: The Uniform Guidelines on Employee Selection Procedures state that a selection rate for any race, sex, or ethnic group which is less than 4/5ths of the rate for the group with the highest passing rate will generally be regarded as evidence of adverse impact (Uniform Guidelines on Employee Selection Procedures, 1978; American Educational Research Association, American Psychological Association, and National Council on Measurement in Education, 1999; Society for Industrial and Organizational Psychology, Inc, 2003). Whilst the 4/5th rule presents a quick measure, the guidelines as well as professional standards recommend statistical measures also be used to establish whether adverse impact is present. We evaluate validity, reliability and adverse impact in line with these psychometric test guidelines and professional testing standards and include tests of proportion or passing rate differences (e.g., Fisher’s exact), and statistical significance tests of group differences (e.g., two standard deviations, and/or Cohen’s *D*).

#### 3.2.3. Results

See Table 4 for practical and statistical measures of Adverse Impact for the game based assessment of cognitive ability described in Study one. The different measures show no adverse impact on any of the evaluated groups. Although the two standard deviations measure exceeds accepted thresholds for female applicants, this test is overpowered when sample sizes exceed 100, which is the case here. Fisher’s exact test is marginally significant, but both the Adverse Impact Ratio and Cohen’s *D* show acceptable levels of group differences between male and female applicants.

**Table 4:** Adverse impact analysis of the game based cognitive ability assessment.

	Protected group	Total	Passing rate	Adverse impact ratio (4/5th rule)	Cohen’s D	2SD	Fisher’s exact test (value of *p*)
Age	Under 40	3,057	67%	0.94	0	0	1
	Over 40	49	63%	1	−0.15	−0.56	0.64
Gender	Female	1,334	65%	0.95	−0.12	−2.11	0.03
	Male	1,575	69%	1	0	0	1
Ethnicity	Asian	815	68%	1	0.08	−0.03	1
	Black	309	61%	0.90	−0.18	−1.02	0.32
	M/N Eastern	66	68%	1	0.02	0	1
	White	1917	67%	0.99	0	−0.11	1

Accepted adverse impact ratio: >0.8. Accepted Cohen’s D: < |0.20|. Accepted 2SD: <|2| but overpowered when *N* > 100. Accepted Fisher’s exact: >0.05. Passing rate is set at the 33rd percentile as a practical illustration of common passing rates used in practise.

#### 3.2.4. Discussion

The adverse impact metrics illustrate the game based cognitive ability assessment falls within acceptable ranges of group differences, even when tested on applicant data that was not used as part of the scoring model generation. That is, the scoring model generates acceptable ranges of group differences when used on new data. This indicates that the game based format and the machine learning based model with Bias Penalisation might have a positive impact on fairness in cognitive ability assessments. However, additional samples are needed to validate the generalisability of the Bias Penalisation to other recruitment contexts, such as other job positions, regions or job levels. In Study three, we look at game mechanisms that might contribute to reducing group differences in game based cognitive ability tests by reducing anxiety.

### 3.3. Study 3: The user experience: Test taking anxiety in game based assessments

A possible avenue to reduce the adverse impact of cognitive ability is reducing test taking anxiety. Test taking anxiety refers to cognitive and behavioural responses accompanying concern about possible negative consequences arising from an evaluative situation (Zeidner, 1998). Test taking anxiety causes poor academic performance (Hembree, 1988; Zeidner, 1998; Cassady and Johnson, 2002), and women tend to experience higher levels of test anxiety {Devine et al., 2012 (maths test); von der Embse et al., 2018 [meta analysis including classroom testing, Grade Point Average (GPA), IQ and standardised exams (e.g., SAT)]}. Game-based assessments may offer a way to combat test anxiety, and its adverse effect on performance. Individuals have positive experiences interacting with games used for serious purposes (Shen et al., 2009). Simple game-like artefacts increase participant enjoyment and interest in activities (Lieberoth, 2015). This study explores whether the gamified cognitive ability assessments result in test taking anxiety, and whether this anxiety impacts performance and does so differently for men and women.

The analysis first tests gender differences in game performance and test taking anxiety experienced as a result of playing the games. Second we use multiple regression to test the effect of test taking anxiety and gender on game performance, as well as the interaction between test taking anxiety and gender on game performance.

#### 3.3.1. Data

The study includes 85 participants recruited from Prolific Academic and compensated for their participation, 52 (61%) are women and 33 (39%) men with a mean age of 34 (SD = 11.45). The sample is predominantly white (86%) and educated beyond highschool (70%). The dataset excludes thirteen additional participants that were removed due to poor response variability, missing answers, or poor game performance indicating no valid attempt.

#### 3.3.2. Measures

##### 3.3.2.1. Test taking anxiety

Test taking anxiety is the difference between an individual’s pre assessment test anxiety and their post assessment test anxiety, with higher test taking anxiety scores indicating lower test taking anxiety after the assessment compared to before. Test anxiety is measured using adapted versions of the Test Anxiety Inventory, answered on a four point Likert scale, from strongly disagree to strongly agree (TAI; Spielberger et al., 1980). TAI measures symptoms of test anxiety experienced before, during, and after testing situations. It has good test–retest and internal-consistency reliability, and acceptable concurrent, construct and discriminant validity (Spielberger et al., 1980; Szafranski et al., 2012; Ali and Mohsin, 2013). Pre assessment test anxiety is measured as a baseline with the five item Test Anxiety Inventory Short Form (TAI-5; Taylor and Deane, 2002). Post assessment test anxiety is measured using an adapted thirteen item version of the TAI (Spielberger et al., 1980), where items referencing future outcomes related to test performance and test feedback are removed, and items are re-worded to measure present test anxiety resulting from the game based assessments. The two inventories are comparable on patterns of correlation, means and standard deviations and show good internal consistency with Chronbach’s Alpha TAI-5 = 0.89; TAI = 0.93 (see Table 5 for descriptives and Table 6 for correlations).

**Table 5:** Means, standard deviations (SD) and group differences between men and women on test taking anxiety and game performance.

	Women	Men	*t*-test (84)	*p*
	Mean (SD)	Mean (SD)		
Test taking anxiety (Pre – Post)	0.07 (0.93)	−0.11 (0.93)	0.91	0.368
TAI-5 (pre)	0.24 (1.02)	−0.37 (0.88)	2.83	0.006**
TAI (post)	0.16 (1.04)	−0.26 (0.91)	1.91	0.059
Games total	−0.21 (2.68)	0.33 (3.36)	−0.91	0.366
Numerosity	−0.13 (0.90)	0.21 (1.14)	−1.52	0.132
Pathfinder	−0.06 (0.99)	0.09 (1.03)	−0.65	0.518
Shapedance	−0.03 (0.99)	0.05 (1.03)	−0.33	0.744
Singularity	0.01 (0.98)	−0.01 (1.05)	0.10	0.917

**p < 0.01.

**Table 6:** Correlations between test taking anxiety and game performance.

	TAI-5 (Pre)	TAI (Post)	Test taking anxiety	Numerosity	Pathfinder	Shapedance	Singularity	Games total
TAI-5 (Pre)	1	0.58**	0.46**	−0.03	0.11	0.08	−0.14	0.00
TAI (Post)	0.58**	1	−0.46**	−0.19	−0.07	−0.17	−0.15	−0.21
Test taking anxiety^+^	0.46**	−0.46**	1	0.18	0.20	0.27*	0.01	0.23*
Numerosity	−0.03	−0.19	0.18	1	0.50**	0.38**	0.30**	0.79**
Pathfinder	0.11	−0.07	0.20	0.50**	1	0.39**	0.30**	0.73**
Shapedance	0.08	−0.17	0.27*	0.38**	0.39**	1	0.44**	0.74**
Singularity	−0.14	−0.15	0.01	0.30**	0.30**	0.44**	1	0.67**
Games total	0.00	−0.21	0.23*	0.79**	0.73**	0.74**	0.67**	1

^+^Higher values on test taking anxiety indicate less anxiety experienced after playing the games compared to before, i.e., a decrease of test taking anxiety compared to the baseline. *p < 0.05. **p < 0.01.

##### 3.3.2.2. Numerosity, pathfinder, shapedance, and singularity game scores

Individual game scores are calculated by multiplying the participants max level achieved by their win ratio.

##### 3.3.2.3. Games total score

Sum of the standardised individual game scores for each of the four games, describing overall game performance.

#### 3.3.3. Results

No significant differences between men and women are observed for performance on the games total score and on individual games. Although women report significantly higher baseline test anxiety before playing the games than men, there are no significant gender differences on test taking anxiety experienced as a result of playing the games (see Table 5).

**Figure 4:**
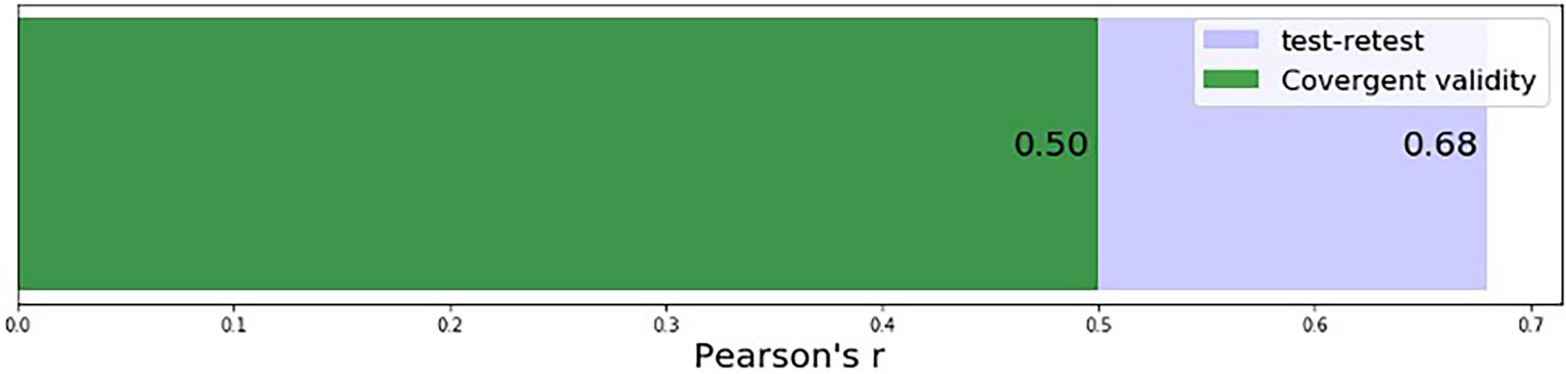
Game performance by anxiety change and gender. A higher test taking anxiety score indicates lower test taking anxiety experienced after playing the games than baseline test taking anxiety measured before playing the games.

There is a significant correlation between test taking anxiety and game performance for the games total score as well as performance on the Shapedance game, such that those who experience less test taking anxiety after playing the games perform better (see Table 6). However, test taking anxiety does not significantly predict game performance: multiple regressions are computed predicting the games total score from test taking anxiety, gender and the interaction of test taking anxiety and gender. The interaction term is included to assess whether test taking anxiety affects men and womens’ performance differently. There are no significant main effects of gender or test taking anxiety on game performance. However, there is a significant interaction effect of gender and test taking anxiety on the games total score. When computing the model for each game individually, the relationship is significant for the Numerosity score only, but not for the remaining game scores. See Table 7 for results of multiple regression models. Interaction plots reveal that men perform better on the games total and Numerosity scores when they experience less test taking anxiety (see Figure 4). Test taking anxiety has no significant effect on women’s performance.

**Table 7:** Multiple regression predicting game performance from gender, test taking anxiety, and the interaction of gender and test taking anxiety.

		Coef	Coef 95% CI [LL, UL]	Std error	*t*(81)	*p*
Games total	Coef	−0.08	[−0.34, 0.18]	0.13	−0.61	0.543
	Gender	0.20	[−0.14, 0.53]	0.17	1.18	0.241
	Test taking anxiety	0.02	[−0.26, 0.31]	0.14	0.16	0.877
	Gender*Test taking anxiety	0.61	[0.15, 1.07]	0.23	2.64	0.010*
Numerosity	Coef	−0.12	[−0.38, 0.13]	0.13	−0.97	0.333
	Gender	0.29	[−0.04, 0.61]	0.16	1.78	0.080
	Test taking anxiety	−0.09	[−0.37, 0.19]	0.14	−0.64	0.524
	Gender*Test taking anxiety	0.79	[0.34, 1.23]	0.22	3.53	0.001**
Pathfinder	Coef	−0.06	[−0.33, 0.21]	0.14	−0.45	0.656
	Gender	0.15	[−0.20, 0.49]	0.17	0.85	0.401
	Test taking anxiety	0.06	[−0.24, 0.35]	0.15	0.40	0.691
	Gender*Test taking anxiety	0.43	[−0.04, 0.91]	0.24	1.81	0.073
Shapedance	Coef	−0.04	[−0.31, 0.23]	0.14	−0.31	0.755
	Gender	0.10	[−0.24, 0.44]	0.17	0.57	0.571
	Test taking anxiety	0.19	[−0.10, 0.49]	0.15	1.30	0.198
	Gender*Test taking anxiety	0.27	[−0.21, 0.74]	0.24	1.13	0.264
Singularity	Coef	0.01	[−0.27, 0.30]	0.14	0.10	0.922
	Gender	0.00	[−0.36, 0.36]	0.18	0.00	0.998
	Test taking anxiety	−0.07	[−0.37, 0.24]	0.15	−0.43	0.668
	Gender*Test taking anxiety	0.19	[−0.31, 0.68]	0.25	0.76	0.450

Gender is coded as male = 1, female = 0. Models predicting the Games total score (*R*^2^ = 0.108, *F*(1, 81) = 4.39, *p* = 0.006) and Numerosity scores (*R*^2^ = 0.161, *F*(1, 81) = 6.39, *p* = 0.0006) are significant, all other models are insignificant. *p < 0.05, **p < 0.01.

#### 3.3.4. Discussion

Women experience higher test taking anxiety at baseline than men, in line with previous studies (Núñez-Peña et al., 2016). However, there is no significant difference in test taking anxiety when adjusting for baseline test taking anxiety, indicating that the games evoke similar levels of anxiety in women and men. As in previous studies showing that test anxiety negatively impacts academic performance, and performance on cognitive ability tests, test taking anxiety correlates with lower game performance in this study (Hembree, 1988; Reeve and Bonaccio, 2008). However, this does not appear to negatively impact women: no gender differences in game performance are observed on the total Games Score or the single games. We also find that test taking anxiety affects the performance of men negatively, in particular on the Numerosity game, but not that of women. This result may be explained with the sex-linked anxiety coping theory (McCarthy and Goffin, 2005). This theory posits that differences in coping strategies mean that although women experience higher levels of test anxiety, they also engage in more coping behaviours (e.g., positive self-talk) than their male counterparts. As a result, the relationship between test anxiety and test performance is weaker for women than men, affecting men’s performance negatively but not women’s.

### 3.4. Study 4: In application: The user experience of job applicants

#### 3.4.1. Data

In this study, we use a dataset of 4,778 job applicants to Graduate roles in a large consulting firm in Eastern Europe. Applicants are typically recent graduates from university and are local to any of the Eastern European countries in scope, which includes countries like Poland, Czech Republic and Slovakia. Demographic data on these applicants is not available due to data protection legislation.

#### 3.4.2. Method

All applicants complete a game-based assessment designed to assess cognitive ability as part of their job application. Applicants apply to technical Graduate roles in the company (e.g., IT roles) and are presented with a cognitive game package that includes three games: Numerosity, Singularity and Shapedance. For applicants in some countries, the game package is combined with an English language test or a business specific technical test.

We collect both quantitative and qualitative data from these applicants. Quantitative data is available from 4,778 candidates, who answer nine questions related to overall satisfaction with the assessment, ease of use, available support and resources for completing the assessment, relevance, and length of the assessment (Table 8 lists all nine areas). The candidates record their answers on a 5-point Likert scale (1 = Highly Dissatisfied; 5 = Highly Satisfied). The candidates also answer the standard Net Promoter Score (NPS) question: “Based on the experience you just had, how likely are you to recommend [Company name] to a friend or colleague?” on a 1–10 scale.

**Table 8:** Descriptive statistics of the user experience questionnaire.

	Mean	SD
Overall satisfaction	4.30	0.77
Ease of use	4.67	0.63
Support and resources	4.57	0.72
Enough preparation time	4.42	0.83
Prepared response	4.38	0.82
Answer time	4.26	0.91
Too many questions	2.30	1.14
Relevant assessment	3.70	0.99
Showcase skills	3.64	1.04

We also collect qualitative comments from candidates using open-ended questions about the assessment experience and whether they have any recommendations or suggestions for improvement. We only include comments in the English language, which amount to 685 usable, substantive comments. These comments are analysed *via* Thematic Analysing (Braun and Clarke, 2006).

#### 3.4.3. Results

The NPS obtained is 58. The NPS scale ranges from-100 to +100, where the percentage of detractors (answering zero to six out of ten) is subtracted from the percentage of promoters (answering nine or ten out of ten). An NPS of 58 indicates a good user experience, comparing favourably to average NPS scores achieved in several industries and product areas (average NPS score 43 in professional services, 35 for technology companies, and 43 for consumer goods companies; Gitlin, 2021).

Table 8 presents the results for the user experience questionnaire. The analysis of the user experience questionnaire also indicates the candidates are overall satisfied with the assessment, they perceive sufficient support and resources, enough preparation time, they are satisfied with the response they had prepared, and they are also satisfied with the answering time provided. The assessment aspects where the applicants show the lowest satisfaction are related to the length of the assessment (i.e., too many questions), the perceived relevance of the assessment, and the degree to which the assessment enabled the applicants to showcase their skills.

The qualitative analyses reveal a number of prevalent themes within candidates’ comments reflecting a positive experience, including positive attitudes (“fun,” “candidate friendly,” “good support,” “more games,” “this is the future”) and overall satisfaction with the format (“everything is perfect as is,” “no further suggestions as everything is good”). At the same time, the analysis reveals several areas of potential improvement for the game-based assessments, including a desire for more practise time to prepare for the assessment and better instructions, and a desire for more technical/job-related content within the assessment.

#### 3.4.4. Discussion

User experience questionnaires obtained from real life job applicants indicate that game based assessments deliver a favourable experience for most candidates. Areas of improvement highlighted both in qualitative and quantitative feedback appear to relate to the abstract nature of cognitive ability tests rather than the game based format: “Relevant assessment” and “showcase skills” ranked lowest in terms of satisfaction, and verbal comments reflected a lack of understanding for the job relevance of the test. This highlights the need to frame assessments in relation to the relevant job competencies they measure, something that may be even more important with game based assessments that have a less serious test-like feel.

## 4. Discussion

With this collection of studies we seek to advance the literature on game based as well as machine learning based assessments, and to highlight the potential of both methodologies to improve the fairness and thereby effectiveness of cognitive ability assessments. We describe and validate a machine learning based scoring algorithm optimised for fairness with good convergent validity (*r* = 0.50), comparable to values described in previous studies ranging from *r* = 0.30 to .93 (McPherson and Burns, 2008; Atkins et al., 2014; Quiroga et al., 2015; Peters et al., 2021). Notably, this is the case when using the novel Bias Penalisation method that optimises the scoring algorithm to minimise group differences at the same time as maximising convergent validity (Rottman et al., 2021, manuscript submitted for publication^1^). This penalisation might have reduced convergent validity, but results suggest validities are comparable to those reported elsewhere without employing penalisation. The resulting assessment provides a valid estimate of cognitive ability that should be accurate enough for the purpose of pre-hire assessments, and at the same time reduces group differences to a level accepted in application. For example, in our illustration in Study 2, adverse impact metrics are within acceptable ranges for selecting out the bottom 33% of applicants, a cut score that is useful for hiring managers in practise.

### 4.1. Generalisability of fairness optimised machine learning scoring algorithm

Like mitigation methods described by assessment providers, the Bias Penalisation is successful at producing a fair assessment in terms of outcome parity (Bogen and Rieke, 2018; Diamond et al., 2020; Leutner et al., 2021; Team PredictiveHire, 2021). However, to our knowledge, Study two is the first to describe the generalisability of a fairness optimised machine learning algorithm for a game based assessments in the scientific literature. The scoring algorithm produced levels of group difference within accepted ranges in a separate sample, providing a first indication that the dual purpose optimization may offer a successful methodology for reducing group differences across samples. However, the two samples are similar in that they were generated from a pool of applications in the same geographical region and with the same job roles and levels represented in both samples. If this finding can be replicated with more heterogeneous samples the described methodology might offer an option for reducing the adverse impact of cognitive ability tests and aligning their use with diversity goals. Generalizability is important in the applied context, as cognitive ability assessments are often used in high volume recruitment by international organisations that use assessments across regions, job levels and functions (Raghavan et al., 2020).

### 4.2. Anxiety and game performance

We found no significant direct prediction of test anxiety for game performance, and no significant gender differences in anxiety produced by the games. This provides additional empirical evidence for claims that gamification can reduce test taking anxiety, as described in previous literature (Mavridis and Tsiatsos, 2017), as well as being a positive indicator for the gender fairness of the games. For women, higher anxiety also did not lead to lower game performance, although this was the case for men on one of the four games tested. The significant relationship for men is of less immediate practical concern given that men tend to be the baseline group with the highest passing rate both on cognitive ability tests as well as the most likely group to be recruited. However, it might highlight potential discrimination for those with test taking anxiety in an assessment context. More studies are needed to replicate this finding and uncover the role of different game mechanics in reducing anxiety, or weakening the link between anxiety and performance.

### 4.3. Applicant reactions

We review applicant feedback showing a good user experience of cognitive ability games in the real world job application context with an NPS score of 58. This is in line with previous studies suggesting the favourable user experience of game based assessments (Guin et al., 2012). However, this data is, to our knowledge, the first describing user experience in the recruitment context, and provides some validation for claims that gamification is beneficial in the job application process. The feedback suggests that game mechanics that induce enjoyment and flow in other areas of application do indeed also produce a favourable game experience in the job selection context. Feedback contained concerns around the perceived applicability of cognitive ability tasks to job performance, an issue commonly raised with cognitive ability tests (Smither et al., 1993; Hausknecht et al., 2004). The face validity aspect of the user experience in game based assessments is not prominently covered in the literature on gamification, but is of particular interest for the recruitment context: Low face validity has a negative effect on applicant perceptions as well as test performance, although the effect on test performance can be mitigated by high test taker motivation, which is likely to occur in job applications (Chan, 1997). Study four illustrates the importance of evaluating gamification in the applied recruitment context.

### 4.4. Impact of technology on cognitive ability testing

With regards to the use of machine learning and game based assessment for the measurement of cognitive ability, this collection of studies raises two broader points: First, that technology can be used to enhance current cognitive ability tests and that its adaptation in practise and research is warranted. And second that the use of these technologies inevitably changes, at least to some degree, what cognitive ability tests measure, and thereby how cognitive ability relates to real life outcomes of interest, including job performance.

Regarding the first point, we argue in this paper that the reduction of group differences and improved user experience are the two technological benefits most relevant in the applied selection context. Game mechanisms are predominantly discussed for their user experience benefits (Landers and Callan, 2011; Huotari and Hamari, 2012). With study three we demonstrate the point that the user experience invoked through gamification has a direct impact on fairness outcomes by dis-or encouraging the participation and performance of applicants from different protected groups. Research on variations in group differences as a result of assessment format exists (Chan and Schmitt, 1997), but has not been explored in the game based assessment space to our knowledge.

Equally, machine learning scoring algorithms are typically discussed as a means to improve fairness (Diamond et al., 2020; Raghavan et al., 2020; Team PredictiveHire, 2021), but they also offer user experience benefits, for example through shorter assessment times (Leutner et al., 2020). Scoring model optimisation methodologies like described in Studies one and two are relatively common in application but rarely described in the academic literature. We hope to provide an applied view of the potential benefits of these scoring methodologies in reducing group differences. They are particularly encouraging as they do not require changing the assessment content itself, a significant practical advantage (Raghavan et al., 2020). However, optimising for outcome parity on known group differences is a narrow view of fairness that does not solve all equality problems in cognitive ability testing. Importantly, optimization can only work on known protected groups. In practise, these are groups that are routinely recorded during the application process such as age, gender, and ethnicity, and that also have large volumes within each group, excluding, for example, smaller ethnic minority groups. Even when mitigated through optimised scoring algorithms, group differences in cognitive ability tests exist. Cognitive ability testing remains a fairness concern and must be carefully balanced with diversity goals when used in application. Factors that influence group differences on cognitive ability tests need to be further explored in the context of applied selection in order to provide a favourable testing environment that is set up for fairness. This includes not only the tests themselves, their framing and face validity, but also the wider recruitment context like job advertisements, employer image, and organisational values and make up.

Regarding the second point, the convergent validity we observe in Study one is high, but variance in gameplay behaviour remains unexplained. New technologies might help deliver fairer and more engaging assessments, but this is only useful in practise if they measure skills and abilities that are relevant at work: The justification for using cognitive ability tests despite the group differences they produce is their consistent relationship with job performance (Schmidt and Hunter, 1998; Kuncel et al., 2010). By losing convergent validity with traditional tests that have high criterion validity, game based assessments might produce a weaker link with job performance. This needs to be tested by relating games to criterion outcomes, and exploring whether unexplained variance relates to job performance. However, even without criterion data, convergent validity as well as content validity, interpreted together with decades of research into the predictive validity of cognitive ability for a range of job roles and level, provide a good foundation to justify the use of game based assessments of cognitive ability in practise. This justification is further strengthened if a game based assessment delivers better outcome parity compared to traditional tests.

### 4.5. Limitations and directions for future work and practise

As a validation and demonstration of game based assessments for the applied selection, this paper has some limitations with regards to concurrent validity: The impact of bias penalisation and gamified format changes on predictive validity for job performance are unclear. There is a clear gap in the literature here that needs to be addressed in future studies. Additionally, each study presents its own limitations. Studies one and two demonstrate that fairness optimised scoring algorithms work on data unseen during scoring model generation. However, further samples from different cultures, languages, employers, and job levels should be explored in order to test the generalisability of the optimised algorithm. This should be done in a structured way to test how optimization responds to data that is different on each of the above characteristics. Tests should additionally include protected groups unknown to the algorithm, such as smaller minority ethnic or neurodiverse groups. Adverse Impact evaluation needs to be an ongoing project for any cognitive ability assessment used in application.

Study three presents clear limitations due to its small sample size and restriction to gender, with ethnic and other protected groups excluded from the analysis. More research is needed to understand the test taking experience of different groups, and how anxiety, stereotype threat, or other characteristics might affect performance on game based assessments. This will help test developers design assessments that produce fewer group differences. The study was also conducted on panellists rather than real world applicants. Given the impact of high versus low stakes settings on test taking behaviour, this research should preferably be conducted in the applied selection context. If possible, test providers’ user experience questionnaires should include measures of test taking anxiety, face validity and other relevant aspects. We want to see more research published on game based assessments used in selection processes.

Study four uses feedback collected from real job applicants, which presents several limitations for analysing and interpreting results. First, some applicants provided feedback directly after playing the games, whilst others took additional tests before providing feedback. This will have affected their responses. Second, applicants did not take any traditional, non-gamified tests and a comparison of their user experience between gamified and non-gamified tests was therefore not possible. We use the Net Promoter score to provide a standardised metric of user experience compared to user experiences in other industries. However, an industry average Net Promoter score is, to our knowledge, not available for psychometric assessments or job applications. We encourage the use of Net Promoter scores in future studies on game based assessments as well as for assessment providers.

## 5. Conclusion

We find that game based assessments of cognitive ability work. They accurately measure cognitive ability, showing good convergent validity with traditional cognitive ability tests, Furthermore, we find that the use of gamification and machine learning based scoring with bias penalisation might help solve the biggest practical issue with cognitive ability tests, their bias, by minimising group differences. This benefit seems to be retained in real world selection applications, at least in the context we are able to evaluate. Anxiety did not impact performance on game based cognitive ability tests, providing some evidence that gamification is beneficial for test taking anxiety. Additionally, we find that the user experience of real world job applicants who take game based assessments of cognitive ability is positive, further supporting their use in applied settings. Gamification did not eliminate face validity concerns of applicants that are typical in cognitive tasks. This is of high practical importance and must be addressed.

Overall, our data show that game based formats and machine learning based scoring deliver key benefits of practical importance, namely increased fairness and user experience. They also offer avenues for the advancement of psychometric test design, for example by allowing the optimisation of scoring keys to fairness outcomes. These new technologies should therefore be investigated and further developed in psychometric testing. Further investigation into their use is encouraged.

## Data availability statement

The raw data supporting the conclusions of this article will be made available by the authors, without undue reservation.

## Ethics statement

The studies involving human participants were reviewed and approved by the Ethics Committee of the Division of Psychology and Language Sciences, Department of Experimental Psychology, University College London. The patients/participants provided their written informed consent to participate in this study.

## Author contributions

FL, S-CC, and TB contributed to the data analysis and write up of Studies 1–4, with TB leading on data analysis. SB and FL conducted Study 4. All authors contributed to the article and approved the submitted version.

## Acknowledgments

We thank the Data Science, Industrial Organizational Psychology, and Product teams at HireVue, as well as Clemens Aichholzer, Luca Boschetti, and Maurizio Attisani for their support and their work developing the game based assessments described in this article.

## Conflict of interest

Authors FL, SB, and TB were employed at HireVue at the time of writing.

The remaining author declares that the research was conducted in the absence of any commercial or financial relationships that could be construed as a potential conflict of interest.

## Publisher’s note

All claims expressed in this article are solely those of the authors and do not necessarily represent those of their affiliated organizations, or those of the publisher, the editors and the reviewers. Any product that may be evaluated in this article, or claim that may be made by its manufacturer, is not guaranteed or endorsed by the publisher.

## Footnotes

^1^Rottman, C., Gardner, C., Liff, J., Mondragon, N., and Zuloaga, L. (2021). New Strategies for Addressing the Diversity-Validity Dilemma with Big Data. J. Applied Psychology.
